# Using Eye-Movement Events to Determine the Mental Workload of Surgical Residents

**DOI:** 10.16910/jemr.11.4.3

**Published:** 2018-08-24

**Authors:** Gonca Gokce Menekse Dalveren, Nergiz Ercil Cagiltay

**Affiliations:** Atilim University, Faculty of Engineering, Department of Software Engineering, Ankara, Turkey

**Keywords:** eye-tracking, eye-movement events, mental workload, task difficulty, surgical virtual environment, endo-neurosurgery

## Abstract

These days, eye-tracking is one of the promising technologies used in different fields such as aviation, arts, sports, psychology and driving for several purposes. Even though it is being used for health purposes, studies involving eye-tracking are rare in the field of endo-neurosurgery. This study aims to use this technology to promote our understanding of the effect related to computer-based instructional materials on mental workload of endo-neurosurgery residents. Four computer-based simulation scenarios are developed based on skill development requirements of endo-neurosurgery residents. Two of them were designed as general models and the other two as simulated surgical models. During these surgery procedures, in real settings, surgical residents need to use their both hands simultaneously to control the endoscope and the operational tool in a coordinated fashion. Therefore, to shed light on the participants’ behaviors, these scenarios are performed with dominant-hand, non-dominant hand and, finally with both-hands using haptic interfaces. Twenty-three residents volunteered in this study. Their eye-movements were recorded while performing the scenarios. According to the results of this study, when performing the simulated surgical models, an increase in the participants’ mental workload was recorded when compared to the other scenarios. Accordingly, it can be concluded that the eye-movements of surgical residents can provide insights about the anticipated level of difficulty about the skill-based tasks. This information might be very critical to properly design and organize instructional materials for endo-neurosurgery, and also to better guide and evaluate the progress of trainees in computer simulation-based skill training environments.

## Introduction

Technology-enhanced educational environment, provide several benefits to improve surgical education programs. For instance, simulation is one of the technologies that allows trainees to perform clinical activities interactively by recreating such operations in a computer-based system without exposing patients to the associated risks (27, 34). However, still there is a need for research to develop strategies for improving the curriculum integration of these systems and for creating standardized approaches. In this respect, the mental workload theory and the eyetracking technology are two important concepts that can be implemented in surgical education programs.

The mental workload concept has long been accepted as an essential aspect of individual performance within complex systems (51). It is reported that mental workload can change the performance of individuals (52) and further affect the competence of the whole system (51). Accordingly, system developers need certain models to assess the mental workload imposed on individuals at an early stages so that alternative system designs can be appraised (51). At the same time, mental workload can negatively affect performance and increase the probability of errors (52), and researchers have spent a great deal of effort developing measures and probes of mental workload (1). Supportively, Moray (33) stated that adjusting the allocation of mental workload could reduce human errors, improve system safety, and increase productivity. In earlier studies, three types of mental workload has been defined: intrinsic load, extraneous or ineffective load, and germane or effective load (44). Intrinsic load is an interaction between the nature of the material being learned and the expertise of the learners (37, 44). Extraneous load is resulting from mainly poorly designed instruction, and germane load is related to processes that contribute to the construction and automation of schemas (37).

Eye-tacking provides a valuable source of information, and events such as fixations, blinks, and pupil diameter can be used to assess the mental workload (47). Accordingly, there are several studies conducted on the assessment of mental workload by using eye-tracking technology (31). A precise evaluation of mental workload will be essential for developing systems that manage user attention (3, 14, 21). Researchers have used eye-movement events found to correlate with cognitive demands (1). For instance, Benedetto et al. (5) examined the changes in blink duration and blink rate in a simple driving task and stated that blink events reflect the effects of visual workload. Another study evaluates the mental workload by developing combined measures based on various physiological indices (41). To determine the mental workload, three physiological signals were recorded; these are: alpha rhythm, eye blink interval, and heart rate variability (41). The study of de Greef, Lafeber, van Oostendorp, and Lindenberg (15) describes an approach for objective assessment of mental workload by analyzing the differences in pupil diameter and several aspects of eye-movement under different levels of mental workload. Eye-movement events are also used in medicine for diagnoses, treatment and training purposes (22) and for clinical applications such as Alzheimer’s (12), HIV-1 infected patients with eye-movement dysfunction (42), and schizophrenia (18). Studies show that these events provide crucial information about how users interact with complex visual displays (29). The field of radiology and visual search (36) and laparoscopic surgery training (25, 46) are among the cases in medicine where eye-tracking approach has been adopted. To provide an example, according to the study by Zheng, Jiang, and Atkins (53), participants perform a simulated laparoscopic procedure, and when the task difficulty is increased, the task completion time and pupil size also increase as a result.

Previous studies were conducted mostly on pupil size changes, but there are other eye-movement events, fixation for example, that can be informative for understanding mental workload. Fixation occurs when eye-movements are nearly still in order to assemble necessary information. Accordingly, in this study fixation number and fixation duration events are used to validate the mental workload imposed by different scenarios. As changes in eye-movement events, such as fixation number and fixation duration, with changes in mental workload are likely affected due to the nature of the scenarios (47), understanding the surgical resident’s mental workload while performing surgical operations is crucial for assessing task difficulties (2). It is stated by Just and Carpenter (23) longer fixation duration related with difficulty in interpreting the information present or a greater involvement in its exploration. Accordingly, it was found that more complex problem results in more fixation numbers and longer fixation duration (4, 31, 38). Also, another study stated that the fixation duration might be related to the mental workload, when the mental workload increases the longer fixation duration for observation occurs (6, 19, 49, 50). Hence, this study attempts to understand the mental workload changes of the participants through their eye-movement events, namely fixation number and fixation duration, while performing tasks having different difficulty levels in four surgical scenarios. Accordingly, the scenarios are developed in different fidelity levels (high- and low-fidelity) which expected to affect mental workload of the participants. Additionally, in each scenario, the hand condition effect on mental workload is also investigated. Hence, in this study it is hypothesized that because of the changes in the mental workload under these situations (different hand conditions, fidelity levels and task difficulties of scenarios) eye-tracking data would display different behaviors. The authors believe that, this information will be very critical to better understand the mental workload of the participants in these situations. This information provides insights to the instructional system designers to better order and adapt related computer-based simulation technologies according to the skill levels and progress of the trainees.

## Methods 

In this experimental study, 23 surgical residents performed the tasks assigned in four different computer-based simulation scenarios by their dominant hand, non-dominant hand and both-hands. During this process, their eyemovement data is recorded by an eye-tracker. The results were analyzed using statistical methods aimed to better understand the surgical residents’ behaviors in these different simulation scenarios.

### Participants 

Twenty three volunteer surgical residents participated in this study from the Neurosurgery and Ear-Nose-Throat (ENT) surgery departments of a medical school. The majority of the participants were male (87.0%) and do not use eye-glasses (73.9%).

### Apparatus 

The eye-movement data of the surgical residents were recorded with an eye-tracker device while the scenarios were performed under different hand conditions with haptic devices. The data was recorded by The Eye Tribe (17) eye-tracker at 60 Hz with a screen resolution of 1920×1080 pixels. The Eye Tribe is a Danish start-up company that produces eye-tracking technology and offers the product to software developers to be incorporated into different applications and programs. The company focuses on a sleek appearance and a portable structure. The Eye Tribe Eye Tracker is an affordable device, thereby making it a potentially available tool for research. According to Coyne and Sibley (11), the Eye Tribe system results are quite promising for human factors researchers. Dalmaijer (13) stated that researchers on a budget can use the Eye Tribe tracker for the evaluation of fixation events and pupil size.

Since haptic devices enable participants to perform movements in the simulated environments, for performing the tasks the Geomagic Touch mid-range professional haptic device (45) is used alongside 3D Systems haptic devices presenting real 3D navigation and force feedback.

### Scenarios 

Four scenarios were developed for the collection of surgical residents’ eye-movement data. These scenarios were implemented using Unity Platform and C# Programming language. The scenarios were performed with the dominant-hand, then with the non-dominant hand and, finally, both-hands in a given fixed period of time. For providing more objectivity, 12 of the participants started to perform the tasks by their dominant hand, and the remaining participants started with their non-dominant hand. Increasing the 3D depth perception, using the surgical instruments efficiently, fast-following up of objects, and improving the ability to plan and strategize were the learning outcomes of these scenarios. Accordingly, different tasks were defined in each scenario to reach, move and control objects in 3D environments simulating real surgical conditions. Current development technologies allow the recreation of real-life operations with adequate fidelity, thus profoundly improving the training environment (34). Accordingly, in this study two of the scenarios were simulated as surgical model and can be considered as higher-fidelity; the other two were based on general models which can be considered as lower-fidelity. High-fidelity scenarios were simulation of a human nose with the view of a real surgical operation and real skin texture. Also the tasks performed in high-fidelity scenarios were more complex than the low-fidelity scenarios. In addition, it is critical for surgical residents to improve their hand skills. In real operations they have to use their both hands in simultaneously. Accordingly, the simulated surgical tasks in this study performed in different hand conditions (dominant, non-dominant and both) to represent different complexity levels of the tasks. Hence, as it is defined the mental load caused by the internal complexity of the learning materials (43), the intrinsic load is expected to be increased in scenarios having higher complexity levels.

**Figure 1.**
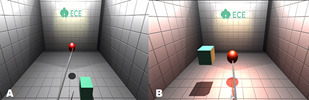
Scenario-1

In Scenario-1, it is necessary to catch the red ball (Figure 1: A) that appears at random places in a room with a surgical tool. After catching the red ball the aim is placing it on the cube, which also appears at random places (Figure 1: B). This scenario is a general simulation model aimed to gain the ability to use the surgical instrument and to develop depth perception and the process has to be completed 10 times in a given fixed period of time.

**Figure 2.**
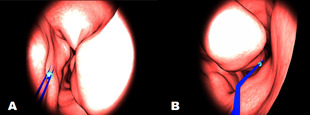
Scenario-2

In Scenario-2, it is necessary to remove the tumor like objects in a given fixed period of time using a surgical tool from a model which was designed based on the inside of a human nose. These tumor objects located in 10 different places (Figure 2: A & B). This scenario is a simulated surgical model, which has made it possible for surgical residents to feel as if they are in surgical settings. Surgical residents can move the endoscopic device through the nose using the haptic device and feel the tissue as the device give force feedback upon collision with any surface. By using the surgical tool in the most accurate way, it is expected to complete the operation by carefully removing the tumors from their locations.

**Figure 3.**
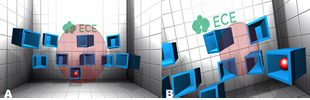
Scenario-3

In Scenario-3, the aim is to approach to the red ball with the correct angle and explode it in a given fixed period of time. This ball appears 10 times in different cubes randomly (Figure 3: A & B). If the correct angle is achieved, the ball will explode; otherwise it will not. In this scenario the aim is to develop depth perceptions and improve ability to approach a certain point with the correct angle. This scenario is a simulation of a general model.

**Figure 4.**
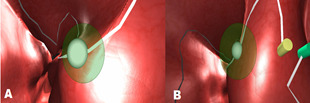
Scenario-4

In Scenario-4, surgical residents are expected to move the ball over a certain path in the nose model by approaching it with a correct angle in a given fixed period of time (Figure 4: A & B). This scenario is a simulated surgical model and designed like inside of human nose with similar texture, simulating the field vision of a surgical resident during an actual operation.

### Procedure 

First, an instruction describing the procedure was given individually and the personal information of the participants were recorded. Volunteers were seated and centered in front of the monitor at a distance of 70cm and 9 calibration points were presented for eye-tracker device calibration. The scenarios were performed in the order of 1, 3, 2 and 4 representing the scenario numbers. Randomly, twelve of the participants started to perform the scenarios first with their dominant-hand and the other group with their non-dominant hand. Afterwards, they performed the tasks with their both hands, under which conditions they used the operation tool with their dominant hand and the camera tool with their non-dominant hand for lighting up the operation area. The recorded raw eye data was classified into number of fixation and fixation duration using an open-source eye-movement classification algorithm (Binocular-Individual Threshold-BIT). BIT algorithm (48) is a velocity-based algorithm to classify fixations from the data with individualspecific thresholds which was implemented in MATLAB. To verify fixations, the algorithm uses the velocity thresholds of both eyes. Also, BIT is a parameter-free fixationidentification algorithm that automatically identifies task- and individual-specific velocity thresholds by optimally exploiting the statistical properties of the eye movement data across different eyes and directions of eye movements (48). The BIT algorithm has advantages over the existing algorithms in that it contains binocular viewing and uses the information about fixations and co-variations between the movements of both eyes to identify saccades; it estimates rather than pre-sets the velocity threshold to identify fixations and saccades, and it permits the threshold to vary between eye-movement directions, tasks and individuals. Also, each record exceeding the threshold value contains the stochasticity which is spontaneous in the eye-movements so as not to be labeled as saccade (48). The other important feature is that BIT algorithm is independent of eye-tracker and sampling frequency and can be easily adapted to the data from varying eye-trackers with different sensitivity and sampling frequency (48). For the evaluation of differences based on scenario difficulties, the fixation number and fixation duration event values were analyzed.

### Measures 

Eye-tracking has been widely used to measure the mental workload from the eye-movement data so as to analyze the cognitive processes underlying visual behavior (47). Eye-tracking provides a valuable source of physiological data for the allocation of information processing resources through ocular activity and are closely linked to the underlying neural networks in the brain (7). To understand the mental workload of surgical residents in these previously explained scenarios, specific measures in eye-tracking were used, namely fixation number and fixation duration events (48). Fixation is a slow period event when the eyemovement is almost still with small dispersion and velocity. With other words eye movements that occur when gaze is dwelling on objects (24). Eye-movement classification algorithms can be able to classify fixation events into number of fixation and fixation duration. Sequences of eye fixations are basic components of eye movements in these fields to gain understanding in visual behavior. Different algorithms have been proposed to identify fixations from the recordings of the point of regard (POR) that the eye tracking equipment provides (48).

## Results 

In all, 276 (23 surgical residents, 4 scenarios, and 3 hand conditions) datasets were recorded, significantly increasing the accuracy of the results in this work. To evaluate and compare the differences among the difficulty levels of the scenarios and hand condition effect, the eyemovement events, fixation number and fixation duration were analyzed.

The analysis of the data was carried out with the SPSS 23 program and it was worked with 95% confidence level. The Friedman non-parametric test technique was used for observing the effect of the difficulty levels within scenarios on the eye-movement events of the surgical residents. To understand the difficulty levels among four scenarios under dominant-, non-dominant and both-hand conditions post hoc analysis with Wilcoxon signed-rank tests was conducted with a Bonferroni correction applied, resulting in a significance level set at p < 0.017.

### Fixation Number 

**Figure 5.**
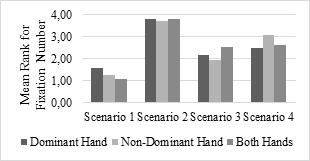
Fixation Number Differences Among Scenarios

A non-parametric Friedman test of differences among the repeated measures was conducted for the scenario difficulty level effect on the fixation number. The effect of the scenario was significant (all ps < .05) on the fixation number according to the results. While the hand condition is fixed, the results of the analysis of the repeated measurements differ according to the scenarios. Based on the Friedman test for different measurement groups, there is a statistically significant difference between the fixation number when using the dominant hand (x^2^ (3) = 37.08, p <0.05) for different scenarios. Scenario-1 has the lowest mean rank for the fixation number (1.57), while Scenario2 has the highest (3.78). When using the non-dominant hand (x^2^ (3) = 50.18, p <0.05) for different scenarios, Scenario-1 has the lowest mean rank for the fixation number (1.26) while Scenario-2 has the highest (3.70) fixation number. According to the test results when using both hands (x^2^ (3) = 52.74, p <0.05) for different scenarios, Scenario-1 has the lowest mean rank for the fixation number (1.07) while Scenario-2 has the highest mean rank (3.80) for the fixation number. According to the results of the three hand conditions for the fixation number measure, the scenario that makes fixation number larger is reported (Figure 5). Generally, in Scenario-2 the fixation number becomes larger compared to the other scenarios.

Wilcoxon signed-rank tests was conducted for understanding the difficulty levels between scenarios under dominant-hand, non-dominant hand and both hands condition with a Bonferroni correction (p < 0.017). The mean and standard deviation values for each scenario under dominant hand, non-dominant hand and both hands conditions are given at Table1.

**Table 1.** Mean and Standard Deviation values for Fixation

Scenario	Dominant Hand		Non-Dominant Hand		Both Hands	
	Mean	Std. Dev.	Mean	Std. Dev.	Mean	Std. Dev.
1	24.66	4.84	26.78	4.11	25.33	5.29
2	55.87	18.18	61.29	21.42	64.40	20.81
3	31.00	9.93	30.54	7.13	46.46	13.48
4	32.36	9.73	46.81	13.09	45.60	25.78

According to the test results there is a significant difference under dominant hand condition between the Scenario-1 and Scenario-2 (Z = -4.14, p = 0.000), Scenario-1 and Scenario-3 (Z = -2.65, p = 0.008), Scenario-1 and Scenario-4 (Z = -3.10, p = 0.002). Similarly, there is a significant difference between Scenario-2 and Scenario-3 (Z = -4.05, p = 0.000) and between Scenario-2 and Scenario-4 (Z = -4.06, p = 0.000). However, the difference between Scenario-3 and Scenario-4 is not statistically significant (Z = -0.68, p = 0.497) under dominant hand condition (Table 2).

**Table 2.** Wilcoxon signed-rank test results (dominant hand)

Scenario	2		3		4	
	Z	p	Z	p	Z	p
1	-4.14	0.000	-2.65	0.008	-3.10	0.002
2			-4.05	0.000	-4.06	0.000
3					-0.68	0.497

According to the test results there is a significant difference under non-dominant hand condition between the Scenario-1 and Scenario-2 (Z = -4.20, p = 0.000), Scenario-1 and Scenario-4 (Z = -4.13, p = 0.000). Similarly, there is a significant difference between Scenario-2 and Scenario-3 (Z = -4.17, p = 0.000), between Scenario-2 and Scenario-4 (Z = -2.71, p = 0.007) and Scemario-3 and Scenario-4 (Z = -3.96, p = 0.000). However, the difference between Scenario-1 and Scenario-3 is not statistically significant (Z = -2.28, p = 0.022) under non-dominant hand condition (Table 3).

**Table 3.** Wilcoxon signed-rank test results (non-dominant hand)

Scenario	2		3		4	
	Z	p	Z	p	Z	p
1	-4.20	0.000	-2.28	0.022	-4.13	0.000
2			-4.17	0.000	-2.71	0.007
3					-3.96	0.000

According to the test results there is a significant difference under both hands condition between the Scenario1 and Scenario-2 (Z = -4.20, p = 0.000), Scenario-1 and Scenario-3 (Z = -4.21, p = 0.000), Scenario-1 and Scenario-4 (Z = -3.97, p = 0.000). Similarly, there is a significant difference between Scenario-2 and Scenario-3 (Z = 3.97, p = 0.000) and between Scenario-2 and Scenario-4 (Z = -3.45, p = 0.001). However, the difference between Scenario-3 and Scenario-4 is not statistically significant (Z = -0.71, p = 0.48) under both hands condition (Table 4).

**Table 4.** Wilcoxon signed-rank test results (both hands)

Scenario	2		3		4	
	Z	p	Z	p	Z	p
1	-4.20	0.000	-4.21	0.000	-3.97	0.000
2			-3.97	0.000	-3.45	0.001
3					-0.71	0.048

### Fixation Duration 

**Figure 6.**
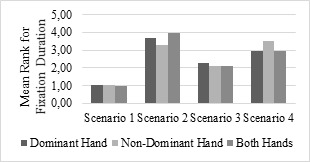
Fixation Duration Differences Among Scenarios

A non-parametric Friedman test of differences among the repeated measures was conducted for the scenario effect on fixation duration (msec.). The effect of scenario was significant (all ps < .05) on the fixation duration according to the results. While the hand condition is fixed, the results of the analysis of the repeated measurements differ according to the scenarios. According to Friedman test for different measurement groups, there is a statistically significant difference between the fixation duration when using the dominant hand (x^2^ (3) = 52.41, p <0.05) for different scenarios. Scenario-1 has the lowest mean rank for the fixation duration (1.04) while Scenario-2 has the highest mean rank for the (3.70) fixation duration. When the non-dominant hand is used (x^2^ (3) = 54.49, p <0.05) for different scenarios, Scenario-1 has the lowest mean rank for the fixation duration (1.04) while Scenario-4 has the highest mean rank for the (3.52) fixation duration. In the both hands condition (x^2^ (3) = 65.56, p <0.05), Scenario-1 has the lowest mean rank for the fixation duration (1.00) while Scenario-2 has the highest mean rank for the (3.96) fixation duration. According to the results of the three hand conditions, the scenario that makes the fixation duration longer is reported (Figure 6). In Scenario-2 and Scenario4 the fixation duration is becomes larger compared to the other scenarios.

Wilcoxon signed-rank tests was conducted for understanding the difficulty levels between scenarios under dominant-hand, non-dominant hand and both hands conditions with a Bonferroni correction (p < 0.017). The mean and standard deviation values for each scenario under dominant hand, non-dominant hand and both hands conditions are given at Table 5.

**Table 5.** Mean and Standard Deviation values for Fixation Duration

	Dominant Hand		Non-Dominant Hand		Both Hands	
Scenario	Mean	Std. Dev.	Mean	Std. Dev.	Mean	Std. Dev.
1	25695.94	5001.86	26697.35	2907.38	32369.00	8352.35
2	59112.81	14626.78	64860.64	16724.14	101785.26	10333.82
3	43239.00	10899.84	44321.84	9344.41	59903.76	9284.96
4	50363.92	12175.10	65791.68	13479.19	77301.20	13700.74

According to the test results there is a significant difference under dominant hand condition between the Scenario-1 and Scenario-2 (Z = -4.19, p = 0.000), Scenario-1 and Scenario-3 (Z = -3.68, p = 0.000), Scenario-1 and Scenario-4 (Z = -4.20, p = 0.000). Similarly, there is a significant difference between Scenario-2 and Scenario-3 (Z = 3.86, p = 0.000) and between Scenario-2 and Scenario-4 (Z = -2.92, p = 0.003). However, the difference between Scenario-3 and Scenario-4 is not statistically significant (Z = -2.16, p = 0.030) under dominant hand condition (Table 6).

**Table 6.** Wilcoxon signed-rank test results (dominant hand)

Scenario	2		3		4	
	Z	p	Z	p	Z	p
1	-4.20	0.000	-4.21	0.000	-3.97	0.000
2			-3.97	0.000	-3.45	0.001
3					-0.71	0.048

According to the test results there is a significant difference under non-dominant hand condition between the Scenario-1 and Scenario-2 (Z = -4.20, p = 0.000), Scenario-1 and Scenario-3 (Z = -4.08, p = 0.000), Scenario-1 and Scenario-4 (Z = -4.20, p = 0.000). Similarly, there is a significant difference between Scenario-2 and Scenario-3 (Z = -3.71, p = 0.000) and between Scenario-3 and Scenario-4 (Z = -4.17, p = 0.000) (Table 7). However, the difference between Scenario-2 and Scenario-4 is not statistically significant (Z = -0.06, p = 0.951) under non-dominant hand condition.

**Table 7.** Wilcoxon signed-rank test results (non-dominant hand)

Scenario	2		3		4	
	Z	p	Z	p	Z	p
1	-4.20	0.000	-4.08	0.000	-4.20	0.000
2			-3.71	0.000	-0.06	0.951
3					-4.17	0.000

According to the test results under both hands condition there is a significant difference between the Scenario1 and Scenario-2 (Z = -4.20, p = 0.000), Scenario-1 and Scenario-3 (Z = -4.21, p = 0.000), Scenario-1 and Scenario-4 (Z = -4.21, p = 0.000). Similarly, there is a significant difference between Scenario-2 and Scenario-3 (Z = 4.21, p = 0.000), between Scenario-2 and Scenario-4 (Z = -3.69, p = 0.000) and between Scenario-3 and Scenario-4 (Z = -4.14, p = 0.000) under both hands condition (Table 8).

**Table 8.** Wilcoxon signed-rank test results (both hand)

Scenario	2		3		4	
	Z	p	Z	p	Z	p
1	-4.20	0.000	-4.21	0.000	-4.21	0.000
2			-3.21	0.000	-3.69	0.000
3					-4.14	0.000

## Discussion 

This research describes an approach for an objective assessment of mental workload by analyzing the differences in the fixation number and fixation duration under different levels of mental workload while surgical residents perform simulated scenarios. The eye-movement data was collected with an eye-tracking device and classified into fixation number and fixation duration events with an eye-movement classification algorithm (BIT). These eye-movement events are selected because they seem to be most suited to provide insight about changes in mental workload (16). There are many other eye-movement classification algorithms, but in this study an open-source eye-movement classification algorithm, BIT, was used. The reason behind this choice was that BIT algorithm is eye-tracker independent and easy to implement and use. The aim of this study is to examine whether the fixation number and fixation duration events can, indeed, be indicators for mental workload and whether there are any among the imposed mental workloads within different scenarios. According to the results, the fixation number and fixation duration both show a significant increase if the mental workload increases. For understanding the differences between the scenarios, four of them were developed in this study; two were simulated surgical models and two were general models. The results can be summarized as highlighted below:

- In the dominant hand condition, Scenario-1 has the lowest mean rank for the fixation number (1.47) and fixation duration (1.04) while Scenario-2 has the highest mean rank for the fixation number (3.78) and fixation duration (3.70).
- When using the non-dominant hand, Scenario-1 has the lowest mean rank for the fixation number (1.26) and fixation duration (1.04), while Scenario-2 has the highest mean rank for fixation number (3.70) and Scenario-4 has the highest mean rank for fixation duration (3.52).
- When using both hands, Scenario-1 has the lowest mean rank for the fixation number (1.07) and fixation duration (1.00), whereas Scenario-2 has the highest mean rank for fixation number (3.80) and fixation duration (3.96).

In general, it can be concluded that in the scenarios that are designed by using the models that simulate the operational area (Scenario 2 & 4), the fixation duration and fixation number values become higher compared to the other group of scenarios (Scenario 1 & 3).

In previous studies, it has been stated that fixation time both show a general significant increase if the mental workload increases (15). Another study stated that the pupil size increased in response to task difficulty (35). Iqbal et al. (21) also stated that more difficult tasks demand longer processing times, induce higher subjective ratings of mental workload, and reliably evoke greater pupillary response at corresponding subtasks than a less difficult task. Additionally, Zheng et al. (53) stated that the pupil size of surgical residents is influenced depending on the task difficulties increasing as the difficulty level elevates. It is also reported that the fidelity level is a crucial factor affecting the mental workload (34). According to the previous studies fixation number and fixation duration are widely used eye-movement events and are generally believed to increase with increasing mental workload (20, 26, 28, 30, 32, 39, 40). In support to these studies, our results show that the scenarios based on simulated tasks using surgical models (higher level of fidelity) increase surgical residents’ mental workloads. Hence, it can be concluded that eye-movement events, such as fixation number and fixation duration, can be used to increase our knowledge of the mental workload of surgical trainees. Since the four scenarios were not performed in randomized and balanced order amongst the surgical residents there might be a training effect. Even this training affect, the results show that lately performed scenarios (2 and 4) are the ones having higher fixation events. Accordingly, this order affect can be considered as acceptable for this study.

Additionally, as there are very limited studies analyzing the eye-movement behaviors of endo-neurosurgery residents, there is no standards in classifying the simulation content according to the level of surgical skills (8, 9). Similarly, the metrics that can be used to evaluate the skill levels of these residents are also very limited and there are no standards on these metrics, either (9). Hence, the results of this study encourage researchers to develop other standardized approaches for using objective metrics in surgical skill performance. Additionally, the results may guide instructional designers to better organize the content of computerbased simulation scenarios through the eye-movement behaviors of the trainees. As reported in the earlier studies, individual characteristics, situational characteristics and training motivation explain incremental variance in training outcomes beyond the effects of cognitive ability (10). These individual differences are more effective in the case of skill-based training environments such as endo-neurosurgery which requires development of both cognitive and psychomotor abilities. By using information collected from the trainees’ behaviors such as eye-movement data, instructional designers can adapt the sequence and difficulty levels of the tasks on each trainee to provide a training opportunity according to the skill and progress levels of each trainee. Hence, in the future the computer-based instructional software developed for skill-based training purposes will be more adaptive by using the data collected from the behaviors (such as eye-movements) and performance of the trainees.

## Ethics and Conflict of Interest 

The author(s) declare(s) that the contents of the article are in agreement with the ethics described in http://biblio.unibe.ch/portale/elibrary/BOP/jemr/ethics.html and that there is no conflict of interest regarding the publication of this paper.

## Acknowledgements 

This study is conducted for improving the scenario designs of the educational materials which are developed for endo-neurosurgery education project (ECE: Tübitak 1001, Project No: 112K287) purposes. The authors would like to thank the support of TÜBİTAK 1001 program for realizing this research. The researchers would also like to thank the ECE project team and the Hacettepe University Medical School for their valuable support throughout the research.
